# Development of a narrow-band imaging classification to reduce the need for routine biopsies of gastric polyps

**DOI:** 10.1093/gastro/goaa080

**Published:** 2020-12-27

**Authors:** Ivor B Asztalos, Caitlin A Colling, Anna M Buchner, Vinay Chandrasekhara

**Affiliations:** 1 Department of Pediatrics, Children’s Hospital of Philadelphia, Philadelphia, PA, USA; 2 Department of Internal Medicine, Massachusetts General Hospital, Boston, MA, USA; 3 Division of Gastroenterology and Hepatology, Hospital of the University of Pennsylvania, Philadelphia, PA, USA; 4 Division of Gastroenterology and Hepatology, Mayo Clinic, Rochester, MN, USA

**Keywords:** gastric polyps, narrow-band imaging, gastric cancer, malignancy, endoscopy

## Abstract

**Background:**

Most incidental gastric polyps identified during upper endoscopy are considered low-risk. However, current guidelines recommend sampling all gastric polyps for histopathologic analysis. We aimed to devise a simple narrow-band imaging (NBI) classification to reduce the need for routine biopsies of low-risk gastric polyps.

**Methods:**

Pairs of NBI and white-light images were collected from 73 gastric polyps for which concurrent histopathologic diagnosis was available. A diagnostic accuracy cohort study was performed. Two blinded endoscopists independently analysed NBI features of each polyp for color, vessel pattern, surface pattern, and any combinations thereof to develop a classification scheme to differentiate low-risk polyps (fundic-gland or hyperplastic) from high-risk polyps (adenomatous or adenocarcinoma) and fundic-gland polyps (FGPs) from non-FGPs.

**Results:**

An isolated lacy vessel pattern and a homogenous absence of surface pattern successfully differentiated low-risk from high-risk gastric polyps. Combining both descriptors into a single algorithm resulted in a negative predictive value (NPV) of 100% [95% confidence interval (CI): 100%–100%], positive predictive value (PPV) of 13.7% (95% CI: 2.6–24.8), sensitivity of 100% (95% CI: 100%–100%), and specificity of 53.7% (95% CI: 45.3%–62.0%) for high-risk polyps. This would reduce the number of polyps requiring biopsy by 50%, while still capturing all high-risk polyps. Regarding FGPs, using a rule not to biopsy polyps with isolated lacy vessels resulted in a 94.9% NPV (95% CI: 89.2%–100%), 63.2% PPV (95% CI: 47.2%–79.2%), 94.8% sensitivity (95% CI: 89.5%–100%), and 63.6% specificity (95% CI: 51.3%–76.0%) for non-FGPs.

**Conclusion:**

In this derivation cohort study, NBI is helpful for differentiating between high-risk and low-risk gastric polyps, thereby reducing the need for routine sampling of low-risk polyps. These results need to be validated in a separate test population.

## Introduction

Gastric epithelial polyps are routinely identified during upper gastrointestinal (GI) endoscopy and encompass a variety of subtypes with distinct histology and malignant potential. In the West, the majority of gastric polyps found during endoscopy are incidental and frequently (70%–94%) are either hyperplastic polyps (HPs) or fundic-gland polyps (FGPs) [1, 2]. A 1-year national study of >120,000 patients in the USA noted the prevalence of gastric polyps to be 6.35%, of which 77% were FGPs and 17% were HPs [1]. Sporadic FGPs may develop in association with long-term use of proton pump inhibitors but have not been associated with an increased risk of cancer [3–5]. It is currently recommended to sample all gastric polyps for histopathologic analysis to guide management decisions, as visual inspection alone using white-light endoscopy (WLE) has been insufficient to differentiate polyps with different histology [2, 6–9]. However, this practice adds considerable cost and utilizes additional resources.

Narrow-band imaging (NBI) is an image-enhanced modality that utilizes a narrow-wavelength light source to optimize hemoglobin light absorption thereby allowing the characterization of microvessels in the mucosa and submucosa, and the mucosal microscopic structure. NBI has been demonstrated to reliably differentiate pre-cancerous small distal colorectal polyps from HPs with no malignant potential using a classification based on color, vessel pattern, and surface pattern [10, 11]. Colonic HPs tend to be lighter or similar in color compared to the background mucosa, have isolated lacy vessels or no vessels present, and have a surface pattern of dark or white uniform spots or an absence of a surface pattern [11]. Conversely, colonic adenomas typically have a browner color relative to the background mucosa, have brown vessels, and a surface pattern with oval, tubular, or branched white structures. In 2011, the American Society for Gastrointestinal Endoscopy (ASGE) released a Preservation and Incorporation of Valuable endoscopic Innovations (PIVI) document regarding a real-time histology assessment of diminutive colorectal polyps, which recommended that, in order for a technology to guide decision-making for the endoscopic management of suspected rectosigmoid HPs of ≤5 mm, it should provide a ≥90% negative predictive value (NPV) for adenomatous histology when interpreted with high confidence [12]. Since then, multiple studies have confirmed the ability to meet this threshold with NBI for colorectal polyps with substantial cost savings and no impact on efficacy [10, 13].

With regard to gastric lesions, small pilot studies have demonstrated that NBI can differentiate intestinal metaplasia from non-neoplastic gastritis [14, 15]. More recently, in a pilot trial from Japan, NBI with magnification endoscopy has been show to accurately predict the histopathology of gastric polypoid lesions based on microvascular patterns [16]. However, the need for magnification endoscopy and the complex classification scheme make it potentially difficult to generalize these findings to other centers. The purpose of this study is to devise a simple descriptive classification for the NBI of small gastric polyps based on existing classification schemes and to evaluate the performance characteristics of those criteria both in isolation and in combination to differentiate benign (FGP and HP) from pre-cancerous or malignant polyps. The primary aim was to devise a classification scheme that would achieve a threshold of ≥90% NPV for high-risk polyps (adenoma or adenocarcinoma), similar to the ASGE PIVI thresholds for colorectal polyps.

## Patients and methods

### Study design

We conducted a diagnostic accuracy cohort study to develop a classification scheme for the endoscopic diagnosis of gastric polyps using NBI. Two NBI-experienced endoscopists participated in the study, both of whom had completed an additional fourth-year advanced endoscopy fellowship and had been in practice for 5–7 years. Prior to study participation, both endoscopists reviewed a library of 100 colorectal-polyp images from 50 polyps that included a high-definition (HD) WLE and NBI image of the same polyp without magnification. The endoscopists had to demonstrate >90% accuracy with the histologic assessment and competency with NBI prior to inclusion in this study.

Images of small (<1-cm) mucosal-based gastric polyps from patients undergoing outpatient esophagogastroduodenoscopy (EGD) between 1 January 2014 and 16 February 2016 were collected from endoscopy reports from a large academic tertiary-care medical center. In live cases, endoscopists assess polyps with both WLE and NBI, and therefore it was decided to include one HD WLE and one NBI image of the same gastric polyp for review for ≤60 seconds. All patients for whom both NBI and WLE images and histology were available were included in the study. Polyps were classified histologically by GI pathologists at the same hospital as either (i) FGP, (ii) HP, (iii) adenomatous polyps, or (iv) adenocarcinoma. Polyps of ≥1 cm, neuroendocrine tumors, and sub-epithelial lesions (e.g. gastrointestinal stromal tumors) were excluded. Patients with hereditary genetic syndromes were also excluded (e.g. familial adenomatous polyposis). The study was approved by the Institutional Review Board of the University of Pennsylvania and conforms with the principles outlined in the the Declaration of Helsinki.

### Descriptors determination and diagnostic-algorithm creation

**Figure 1.**
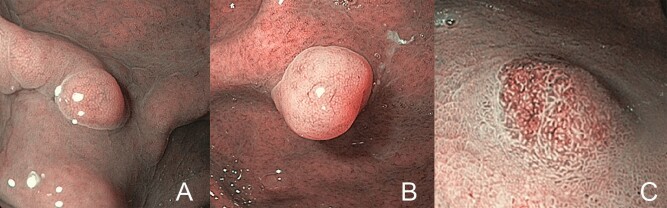
Narrow-band imaging (NBI) color classification. (A) Same as background. (B) Lighter than background. (C) Brown relative to background.

**Figure 2.**
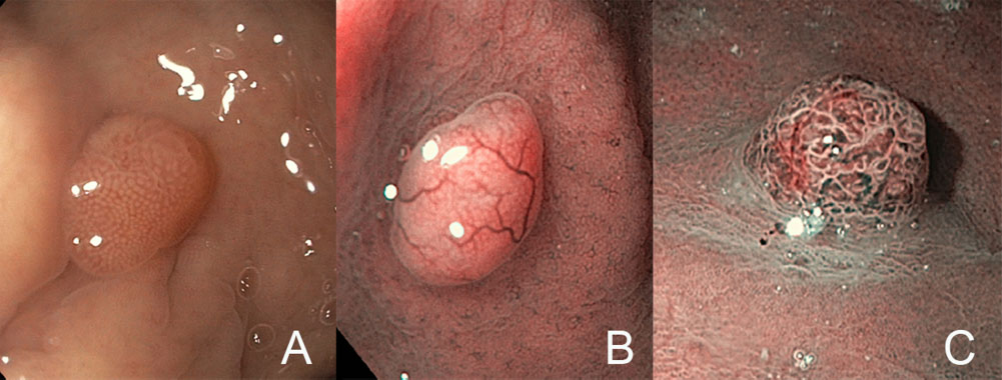
Narrow-band imaging (NBI) vessel classification. (A) No vessels. (B) Isolated lacy vessels. (C) Brown vessels.

**Figure 3.**
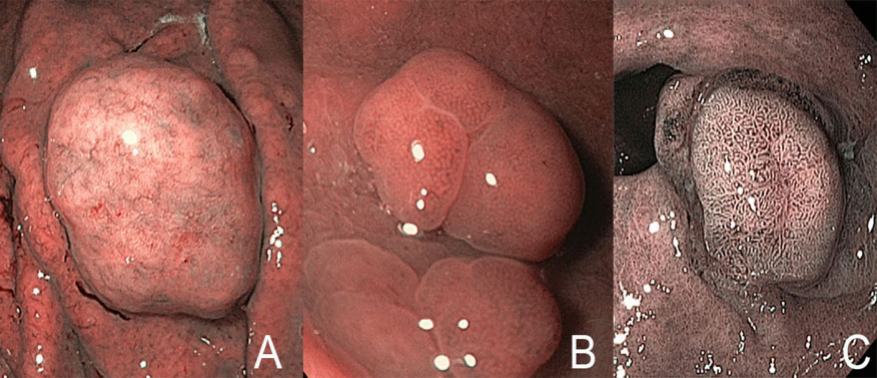
Narrow-band imaging (NBI) surface-pattern classification. (A) Homogenous absence of pattern. (B) White spots of uniform size. (C) Branched white structures surrounded by brown vessels.

Using nomenclature from the NBI International Colorectal Endoscopic classification as a guide [11], three descriptors were chosen to describe the gastric lesions: color—same as/lighter than background or browner relative to background (**Figure 1**); vessel pattern—no vessels, isolated lacy vessels, or brown vessels (**Figure 2**); and surface pattern—homogenous absence of pattern; dark or white spots of uniform size; and oval, tubular, or white structures surrounded by brown vessels (**Figure 3**). Both endoscopists independently evaluated the NBI images based on these three characteristics that, in isolation and in combinations thereof, were evaluated on their ability to correctly identify gastric lesions that were of low risk and did not require biopsy—defined, for the purposes of this study, as either FGP or HP.

### Measured outcomes

The primary outcome of this derivation cohort study was to develop an algorithm that maximizes the NPV for identifying low-risk polyps (FGP and HP) vs high-risk polyps (adenoma and adenocarcinoma). The discriminatory capacity of the individual NBI characteristics to predict all non-FGPs from FGPs was a secondary outcome.

### Statistical analysis

Point estimates and the 95% confidence intervals (CIs) for NPV, positive predictive value (PPV), sensitivity, specificity, and positive and negative likelihood ratios (LRs) were calculated for each endoscopist and for their combined performance for the prediction of low-risk polyps. Point estimates of these parameters for the individual endoscopists were calculated in the traditional way and exact binomial CIs were calculated. As the same polyps were evaluated by both endoscopists in the combined results, methods to analyse dichotomous diagnostic tests with clustered data were used with clustering at the level of the polyp for the NPVs, PPVs, sensitivities, and specificities [17].

## Results

### Characteristics of lesions and image sets

A total of 73 small gastric polyps were identified in the study period with at least one HD WLE and NBI image. Histology demonstrated 44 FGPs (60.3%), 24 HPs (32.9%), 3 adenomas (4.1%), and 2 adenocarcinomas (2.7%). A representative type of each image was selected to construct 73 pairs of images, which were reviewed by two expert endoscopists, thereby leading to 146 reviewed NBI images of polyps. NBI assessment of each image feature with regard to color, vessel pattern, and surface pattern are listed in **Table 1**.

**Table 1.** Image interpretation of the 146 narrow-band-imaging (NBI) images of 73 gastric polyps compared to the histologic assessment of high-risk and low-risk polyps

NBI feature	High-risk polyp (*n* = 10)	Low-risk polyp (*n* = 136)
Color		
Lighter or similar	3	81
Browner	7	55
Vessel pattern		
Absent	3	35
Isolated, lacy	0	59
Brown	7	42
Surface pattern		
Absent	0	44
Uniform spots	2	43
Oval, tubular, or branched	8	49

### NBI-algorithm creation for differentiating low-risk from high-risk polyps

**Figure 4.**
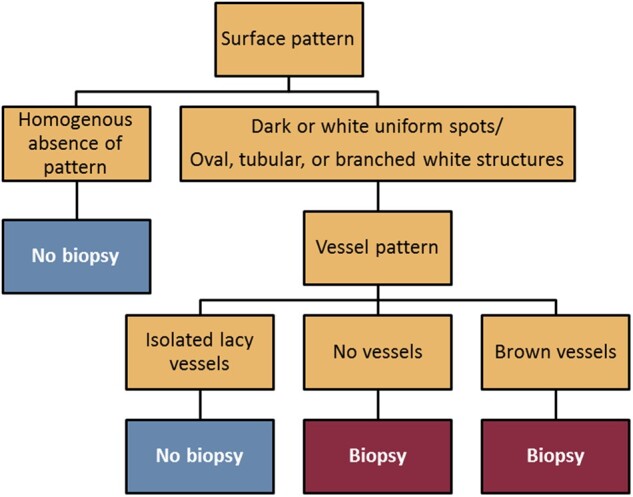
Final algorithm for differentiating high-risk from low-risk gastric polyps based on 146 narrow-band imaging (NBI) images

Due to the low rate of high-risk lesions amongst all gastric polyps and the cost of missing even a single high-risk lesion, the decision was made to utilize only those algorithms that had a 100% NPV for differentiating low-risk from high-risk polyps. **Table 1** highlights the NBI interpretations of polyps compared to the histologic assessment of low-risk and high-risk polyps. The algorithm that identified the largest number of low-risk polyps and retained a 100% NPV entailed only two criteria: polyps that had a homogenous absence of surface pattern *or* those that had isolated lacy vessels (**Figure 4**).

This two-step algorithm safely identified half of the polyps as being low-risk: 36 and 37 for each endoscopist (**Table 2**). For each of the endoscopists individually and for their combined performance, this resulted in an NPV of 100% for high-risk polyps (**Table 3**). Each of the endoscopists classified all five of the high-risk polyps as not being low-risk, demonstrating a sensitivity of 100%. As a result, a low-risk polyp designation from the algorithm entirely ruled out the possibility of pathology with a negative LR of 0, again without bounds. Mirroring the conservativeness of the PPV, the specificity of the algorithm was 53.7% (95% CI: 45.3%–62.0%) with a positive LR of 2.16 (95% CI: 1.83–2.63). The algorithm also performed better than the endoscopists gestalt predictions (**Supplementary Table 1**), in which both endoscopists mislabeled an adenoma and an adenocarcinoma, respectively, as an hyperplastic polyp resulting in an NPV of 80% based on endoscopic opinion alone.

**Table 2.** Endoscopist determination of high-risk vs low-risk gastric polyps based on the devised narrow-band-imaging (NBI) classification

Endoscopist determination	All polyps (*n* = 73)	Fundic-gland polyp (*n* = 44)	Hyperplastic polyp (*n* = 24)	Adenoma (*n* = 3)	Adenocarcinoma (*n* = 2)
Endoscopist A					
High-risk polyp	37	12	20	3	2
Low-risk polyp	36	32	4	0	0
Endoscopist B					
High-risk polyp	36	9	22	3	2
Low-risk polyp	37	35	2	0	0
Combined A and B					
High-risk polyp	73	21	42	6	4
Low-risk polyp	73	67	6	0	0

**Table 3.** Performance of individual endoscopists and combined performance utilizing the devised narrow-band-imaging (NBI) classification for differentiating high-risk from low-risk gastric polyps

Performance	Endoscopist A	Endoscopist B	Combined
Negative predictive value, % (95% CI)	100.0 (90.3–100.0)	100.0 (90.5–100.0)	100.0 (100.0–100.0)
Positive predictive value, % (95% CI)	13.7 (4.5–28.8)	13.9 (4.7–29.5)	13.7 (0.0–27.3)
Sensitivity, % (95% CI)	100.0 (47.8–100.0)	100.0 (47.8–100.0)	100.0 (100.0–100.0)
Specificity, % (95% CI)	52.9 (40.4–65.2)	54.4 (41.9–66.5)	53.7 (45.3–62.0)
Positive likelihood ratio (95% CI)	2.13 (1.65–2.73)	2.19 (1.69–2.84)	2.16 (1.83–2.63)
Negative likelihood ratio (95% CI)	0.0 (0.0–0.0)	0.0 (0.0–0.0)	0.0 (0.0–0.0)

CI, confidence interval.

### NBI algorithm for differentiating FGPs from all other polyps

As virtually all sporadic FGPs require neither follow-up nor biopsy due to the exceedingly low rate of malignancy arising from FGPs, we examined the ability of the descriptors to predict this specific type of polyp. Of the three descriptors used, vessel pattern proved the most effective at differentiating FGPs from all other lesions, including HPs. Of 59 NBI images described as having isolated lacy vessels, 56 were FGPs, yielding an NPV of 94.9% (95% CI: 89.2%–100%) for non-FGPs (**Table 4**). The three additional polyps described as having isolated lacy vessels were found to be HPs. Neither color nor surface pattern achieved the 90% NPV cut-off.

**Table 4.** Negative predictive values (95% confidence interval) for determining non-fundic-gland polyps (FGPs) based on narrow-band-imaging (NBI) characteristics

NBI feature	Endoscopist A	Endoscopist B	Combined
Color	82.0 (68.6–91.4)	94.1 (80.3–99.3)	86.9 (78.8–95.0)
Vessel pattern	93.5 (78.6–99.2)	96.4 (81.7–99.9)	94.9 (89.2–100.0)
Surface pattern	83.0 (69.2–92.4)	88.1 (74.4–96.0)	85.4 (75.9–94.8)

Color: Similar to or lighter than background mucosa (FGP) vs Browner than background (non-FGP).

Vessel pattern: Isolated lacy vessels (FGP) vs No vessels or Brown vessels (non-FGP).

Surface pattern: Homogenous absence of pattern or Dark or white uniform spots (FGP) vs Oval, tubular, or branched structures (non-FGP).

## Inter-observer agreement

Agreement over both the individual descriptors and the diagnostic algorithm ranged from moderate to substantial. The endoscopists agreed on which polyps were low-risk using the overall diagnostic algorithm 79% of the time (*κ*  =  0.59, *P *<* *0.001). Of the individual descriptors, vessel pattern demonstrated the highest agreement (*κ*  =  0.63) while color and surface pattern were similar (κ  =  0.47 and 0.50, respectively); all three were statistically significant (all *P *<* *0.001).

## Discussion

Gastric polyps are commonly found during upper GI endoscopy. Polyps of ≥1 cm in size are generally recommended for resection due to the increased risk of advanced neoplasia [2, 7]. However, the majority of gastric polyps encountered during endoscopy in the USA are incidental findings measuring ≤5 mm and represent benign pathology with virtually no malignant potential. Yet, multiple experts have recommended tissue sampling in all patients with gastric polyps, regardless of the size or appearance, to confirm histological subtype [2, 7, 8]. Small FGPs and HPs are considered benign lesions and are generally not recommended for endoscopic resection. HPs rarely undergo neoplastic progression and, if they do, they are usually polyps that are >1 cm in size. However, HPs are associated with an increased risk of synchronous cancer and therefore, if a polyp is suspected of being an HP, the surrounding gastric mucosa should be carefully inspected [7].

Given that the rate of dysplasia in sporadic FGPs is <1% and that FGPs have not been associated with gastric neoplasia [4, 7], there has been effort to find endoscopic features that reliably predict FGPs without the need for biopsy. A study of 56 patients with gastric polyps who underwent EGD concluded that FGPs can be diagnosed by endoscopic appearance alone with an accuracy of 89% [18]. However, this prior study failed to reach the 90% NPV threshold established by the ASGE PIVI for colorectal polyps [12].

Based on the performance characteristics of NBI for small colorectal polyps and that the majority of gastric polyps are benign, we hypothesized that NBI would be able to identify low-risk lesions that would support a ‘diagnose-and-leave’ strategy for many small gastric polyps. Due to the low overall prevalence of pathologic polyps in this study, which reflects real-world practice in the USA, an algorithm that simply did not biopsy any of the lesions would have reached an NPV of 93% for the primary aim, effectively clearing the recommended threshold suggested by the ASGE PIVI. With 146 NBI images of 73 polyps reviewed by two endoscopists, the 95% CI of this estimate would have crossed the 90% threshold but the uncertainty in this case would be due not to the algorithm, but to the variance around the sample estimate of the rate of pathologic polyps. As such, only algorithms that achieved an NPV of 100% were deemed acceptable for differentiating low-risk from high-risk polyps. Given that the current standard of practice is to biopsy *all* gastric polyps, any reduction in this rate, even by conservative algorithms, would be beneficial. Ultimately, some of the criteria proved insufficiently reliable to differentiate high-risk from low-risk polyps based on our goal NPV of 100%. Notably, color was incapable of differentiating between benign and neoplastic polyps. Of the remaining descriptors, isolated lacy vessels and homogenous absence of a surface pattern reached sufficient diagnostic utility for identifying low-risk polyps.

The ability of the algorithm to avoid the biopsy of low-risk lesions was driven primarily by its identification of FGPs, as the algorithm would have suggested sampling most HPs. Therefore, we investigated the same characteristics to determine whether they were also useful in differentiating FGPs from all other lesions. Ultimately, only the presence of isolated, lacy vessels cleared the 90% NPV threshold for differentiating FGPs. This result is quite promising in light of the prevalence of FGPs (60%), as chance alone would not clear the established 90% NPV threshold. Furthermore, inter-observer agreement was highest for vessel pattern, suggesting that this may provide a simple, reliable method for excluding non-FGPs and therefore obviate the need for tissue sampling.

Assuming that 94% of encountered gastric polyps during upper endoscopy are FGPs or HPs, and half of these can reliably be excluded from biopsies based on NBI-imaging features, we estimate that the routine implementation of this simple technique will eliminate the need for additional tissue sampling in ≤47% of endoscopies with gastric polyps in the Western population [1]. Assuming that 6.35% of endoscopies identify polyps, this could potentially eliminate the need for tissue sampling in ≤3% of all upper-endoscopic procedures in the USA, which could translate into substantial cost savings to health systems [1].

Of considerable strength to this study was the use of a gold-standard reference, histopathologic diagnosis for each and every polyp. The final algorithm’s usage of simple NBI descriptive criteria without the need for magnification endoscopy also leaves us optimistic that the safe use of this technology can quickly and easily be taught to current gastroenterologists and trainees. This pilot study’s chief limitations are its small size and retrospective nature. NBI is being more commonly used for the evaluation of GI polyps but, at the time of data collection, identifying gastric polyps with both WLE and NBI images was challenging, limiting the total number of polyps to 73. Having more than one endoscopist to evaluate the same set of images mitigated this limitation and increased the generalizability to different gastroenterologists. The former was limited by and controlled for in the clustered nature of the data, but this did limit the interpretation of some boundless CIs. Another limitation is that the study included a limited number of high-risk polyps, mirroring a real-world scenario in the Western hemisphere. Some lesions were only sampled and not completely removed. Therefore, it is possible that some of the incompletely resected polyps contained areas of higher-grade dysplasia that were not histopathologically reviewed. However, this would be a less likely scenario, as we only included small polyps (<1 cm) where even histology from biopsy sampling is more likely to represent the entire pathology of the polyp. In addition, several of the images were originally collected by the evaluating endoscopists. This bias was limited by using anonymized images in a random order and by the lapse of time—in some cases multiple years—between acquisition and evaluation, making specific case recall less likely. Lastly, all of the patients, polyps, and images were tested and collected within a single academic tertiary medical center by NBI-experienced endoscopists, possibly limiting its generalizability and biasing the prevalence of pathologic polyps.

This study demonstrates the potential for NBI to be a useful tool in the real-time endoscopic differentiation of benign from pathologic gastric polyps. Using a simple algorithm indicating not to biopsy gastric polyps that have either a homogenous absence of surface pattern or isolated lacy vessels, endoscopists were able to safely avoid biopsy in about half of gastric lesions whilst still maintaining an NPV of 100%. A validation study is needed to confirm the use of this novel NBI algorithm before it can be implemented in patient care.

## Supplementary data

Supplementary data is available at *Gastroenterology Report* online.

## Authors’ contributions

V.C. was the guarantor and designed the study and performed data acquisition, data analysis and interpretation, drafting, reviewing, and final approval of the article. I.A., C.C., and A.B. contributed to the study design, data analysis and interpretation, drafting, reviewing, and final approval of the article.

## Funding

No funding source.

## Supplementary Material

goaa080_Supplementary_Data

## References

[1] Carmack SW , Genta RM, Schuler CM et al The current spectrum of gastric polyps: a 1-year national study of over 120,000 patients. Am J Gastroenterol 2009;104:1524–32. 19491866 10.1038/ajg.2009.139

[2] Evans JA , Chandrasekhara V, Chathadi KV et al The role of endoscopy in the management of premalignant and malignant conditions of the stomach. Gastrointest Endosc 2015;82:1–8. 25935705 10.1016/j.gie.2015.03.1967

[3] Cristallini EG , Ascani S, Bolis GB. Association between histologic type of polyp and carcinoma in the stomach. Gastrointest Endosc 1992;38:481–4. 1511825 10.1016/s0016-5107(92)70481-7

[4] Genta RM , Schuler CM, Robiou CI et al No association between gastric fundic gland polyps and gastrointestinal neoplasia in a study of over 100,000 patients. Clin Gastroenterol Hepatol 2009;7:849–54. 19465154 10.1016/j.cgh.2009.05.015

[5] Zelter A , Fernandez JL, Bilder C et al Fundic gland polyps and association with proton pump inhibitor intake: a prospective study in 1,780 endoscopies. Dig Dis Sci 2011;56:1743–8. 21127978 10.1007/s10620-010-1493-x

[6] Enestvedt BK , Chandrasekhara V, Ginsberg GG. Endoscopic ultrasonographic assessment of gastric polyps and endoscopic mucosal resection. Curr Gastroenterol Rep 2012;14:497–503. 23001857 10.1007/s11894-012-0292-2

[7] Goddard AF , Badreldin R, Pritchard DM, on behalf of the British Society of Gastroenterology et al The management of gastric polyps. Gut 2010;59:1270–6. 20675692 10.1136/gut.2009.182089

[8] Shaib YH , Rugge M, Graham DY et al Management of gastric polyps: an endoscopy-based approach. Clin Gastroenterol Hepatol 2013;11:1374–84. 23583466 10.1016/j.cgh.2013.03.019 PMC3962745

[9] Beg S , Ragunath K, Wyman A et al Quality standards in upper gastrointestinal endoscopy: a position statement of the British Society of Gastroenterology (BSG) and Association of Upper Gastrointestinal Surgeons of Great Britain and Ireland (AUGIS). Gut 2017;66:1886–99. 28821598 10.1136/gutjnl-2017-314109 PMC5739858

[10] Abu Dayyeh BK , Thosani N, Konda V et al ASGE Technology Committee systematic review and meta-analysis assessing the ASGE PIVI thresholds for adopting real-time endoscopic assessment of the histology of diminutive colorectal polyps. Gastrointest Endosc 2015;81:502–e501–2. e516. 25597420 10.1016/j.gie.2014.12.022

[11] Hewett DG , Kaltenbach T, Sano Y et al Validation of a simple classification system for endoscopic diagnosis of small colorectal polyps using narrow-band imaging. Gastroenterology 2012;143:599–607. e591. 22609383 10.1053/j.gastro.2012.05.006

[12] Rex DK , Kahi C, O'Brien M et al The American Society for Gastrointestinal Endoscopy PIVI (Preservation and Incorporation of Valuable Endoscopic Innovations) on real-time endoscopic assessment of the histology of diminutive colorectal polyps. Gastrointest Endosc 2011;73:419–22. 21353837 10.1016/j.gie.2011.01.023

[13] Hassan C , Pickhardt PJ, Rex DK. A resect and discard strategy would improve cost-effectiveness of colorectal cancer screening. Clin Gastroenterol Hepatol 2010;8:865–9.e861–3. 20621680 10.1016/j.cgh.2010.05.018

[14] Uedo N , Ishihara R, Iishi H et al A new method of diagnosing gastric intestinal metaplasia: narrow-band imaging with magnifying endoscopy. Endoscopy 2006;38:819–24. 17001572 10.1055/s-2006-944632

[15] Bansal A , Ulusarac O, Mathur S et al Correlation between narrow band imaging and nonneoplastic gastric pathology: a pilot feasibility trial. Gastrointest Endosc 2008;67:210–6. 18226682 10.1016/j.gie.2007.06.009

[16] Omori T , Kamiya Y, Tahara T et al Correlation between magnifying narrow band imaging and histopathology in gastric protruding/or polypoid lesions: a pilot feasibility trial. BMC Gastroenterol 2012;12:17. 22356674 10.1186/1471-230X-12-17 PMC3310780

[17] Kwak M , Um SW, Jung SH. Comparison of operational characteristics for binary tests with clustered data. Stat Med 2015;34:2325–33. 25801180 10.1002/sim.6485 PMC4632652

[18] Weston BR , Helper DJ, Rex DK. Positive predictive value of endoscopic features deemed typical of gastric fundic gland polyps. J Clin Gastroenterol 2003;36:399–402. 12702980 10.1097/00004836-200305000-00007

