# Supplementary material for: Development of a narrow-band imaging classification to reduce the need for routine biopsies of gastric polyps
Source: Gastroenterol Rep (Oxf). 2020 Dec 27;9(3):219–25. doi: 10.1093/gastro/goaa080 (PMC8309680; doi:10.1093/gastro/goaa080)
Supplement: goaa080_Supplementary_Data [file goaa080_supplementary_data.docx]

**Supplementary Table 1.** NBI characteristics, endoscopists’ gestalt prediction, recommendation for tissue sampling based on the devised NBI algorithm, and final histology diagnosis for all polyps.

|  | Color | | Vessel Pattern | | Surface Pattern | | Endoscopist Prediction not using algorithm | | Biopsy based on algorithm | | Histology |
| --- | --- | --- | --- | --- | --- | --- | --- | --- | --- | --- | --- |
| No. | 1 | 2 | 1 | 2 | 1 | 2 | 1 | 2 | 1 | 2 |  |
| 1 | Same | Same | No vessels | No vessels | Spots | Absent | FGP | HP | Yes | No | FGP |
| 2 | Brown | Brown | Brown | Brown | Ov/Tu/Br | Ov/Tu/Br | HP | Adenoma | Yes | Yes | HP |
| 3 | Same | Same | No vessels | Iso lacy | Ov/Tu/Br | Absent | FGP | HP | Yes | No | FGP |
| 4 | Same | Same | Iso lacy | Iso lacy | Absent | Spots | HP | FGP | No | No | FGP |
| 5 | Brown | Brown | No vessels | No vessels | Spots | Spots | HP | HP | Yes | Yes | HP |
| 6 | Same | Same | No vessels | Iso lacy | Absent | Absent | FGP | FGP | No | No | FGP |
| 7 | Same | Same | Iso lacy | Iso lacy | Spots | Spots | FGP | FGP | No | No | FGP |
| 8 | Brown | Brown | No vessels | No vessels | Ov/Tu/Br | Ov/Tu/Br | HP | HP | Yes | Yes | HP |
| 9 | Same | Brown | Iso lacy | No vessels | Absent | Absent | FGP | HP | No | No | HP |
| 10 | Same | Same | No vessels | No vessels | Spots | Absent | HP | HP | Yes | No | FGP |
| 11 | Same | Brown | No vessels | No vessels | Ov/Tu/Br | Ov/Tu/Br | HP | Adenoma | Yes | Yes | FGP |
| 12 | Brown | Same | No vessels | Brown | Ov/Tu/Br | Ov/Tu/Br | Adenoma | Adenoma | Yes | Yes | Adenoma |
| 13 | Same | Same | Iso lacy | Iso lacy | Absent | Absent | FGP | FGP | No | No | FGP |
| 14 | Brown | Brown | Brown | Brown | Spots | Spots | HP | HP | Yes | Yes | HP |
| 15 | Brown | Brown | No vessels | Iso lacy | Spots | Ov/Tu/Br | HP | HP | Yes | No | FGP |
| 16 | Same | Same | No vessels | No vessels | Spots | Spots | HP | HP | Yes | Yes | FGP |
| 17 | Brown | Brown | Brown | Brown | Ov/Tu/Br | Ov/Tu/Br | Adenoma | Adenoma | Yes | Yes | Adenoma |
| 18 | Brown | Brown | Brown | Brown | Ov/Tu/Br | Ov/Tu/Br | AdenoCA | AdenoCA | Yes | Yes | AdenoCA |
| 19 | Same | Brown | Brown | Brown | Ov/Tu/Br | Ov/Tu/Br | HP | Adenoma | Yes | Yes | Adenoma |
| 20 | Same | Same | Iso lacy | Iso lacy | Spots | Absent | FGP | FGP | No | No | FGP |
| 21 | Brown | Brown | Brown | Brown | Absent | Ov/Tu/Br | HP | Adenoma | No | Yes | HP |
| 22 | Same | Brown | No vessels | No vessels | Spots | Spots | HP | Adenoma | Yes | Yes | AdenoCA |
| 23 | Same | Same | Iso lacy | Iso lacy | Spots | Spots | FGP | FGP | No | No | FGP |
| 24 | Same | Same | No vessels | No vessels | Spots | Spots | FGP | FGP | Yes | Yes | FGP |
| 25 | Same | Same | Iso lacy | Iso lacy | Absent | Absent | FGP | FGP | No | No | FGP |
| 26 | Same | Brown | Iso lacy | Brown | Ov/Tu/Br | Ov/Tu/Br | FGP | Adenoma | No | Yes | FGP |
| 27 | Same | Same | Iso lacy | Iso lacy | Absent | Absent | FGP | FGP | No | No | FGP |
| 28 | Same | Same | Iso lacy | Iso lacy | Spots | Spots | FGP | FGP | No | No | FGP |
| 29 | Same | Same | Iso lacy | Iso lacy | Spots | Absent | FGP | FGP | No | No | FGP |
| 30 | Brown | Brown | Brown | Brown | Ov/Tu/Br | Ov/Tu/Br | HP | Adenoma | Yes | Yes | HP |
| 31 | Same | Brown | No vessels | Brown | Ov/Tu/Br | Ov/Tu/Br | HP | Adenoma | Yes | Yes | HP |
| 32 | Brown | Brown | No vessels | Brown | Ov/Tu/Br | Ov/Tu/Br | HP | Adenoma | Yes | Yes | HP |
| 33 | Brown | Brown | Brown | Brown | Ov/Tu/Br | Ov/Tu/Br | Adenoma | Adenoma | Yes | Yes | FGP |
| 34 | Brown | Brown | Brown | Brown | Ov/Tu/Br | Ov/Tu/Br | HP | HP | Yes | Yes | HP |
| 35 | Brown | Brown | Brown | Brown | Ov/Tu/Br | Ov/Tu/Br | HP | Adenoma | Yes | Yes | HP |
| 36 | Same | Same | Iso lacy | Iso lacy | Absent | Absent | FGP | FGP | No | No | FGP |
| 37 | Same | Same | Iso lacy | Iso lacy | Spots | Absent | FGP | HP | No | No | FGP |
| 38 | Brown | Brown | Brown | Brown | Absent | Ov/Tu/Br | HP | AdenoCA | No | Yes | HP |
| 39 | Same | Brown | No vessels | Iso lacy | Ov/Tu/Br | Spots | HP | HP | Yes | No | FGP |
| 40 | Same | Same | Iso lacy | Iso lacy | Absent | Spots | FGP | FGP | No | No | FGP |
| 41 | Same | Brown | No vessels | No vessels | Spots | Ov/Tu/Br | HP | Adenoma | Yes | Yes | HP |
| 42 | Same | Same | Iso lacy | Iso lacy | Absent | Spots | FGP | FGP | No | No | FGP |
| 43 | Same | Same | Iso lacy | Iso lacy | Absent | Spots | FGP | FGP | No | No | FGP |
| 44 | Same | Same | Iso lacy | No vessels | Spots | Ov/Tu/Br | FGP | Adenoma | No | Yes | FGP |
| 45 | Brown | Same | No vessels | No vessels | Absent | Absent | HP | FGP | No | No | FGP |
| 46 | Same | Same | Iso lacy | Iso lacy | Spots | Spots | FGP | HP | No | No | FGP |
| 47 | Same | Same | Iso lacy | Iso lacy | Spots | Spots | FGP | HP | No | No | FGP |
| 48 | Same | Same | Iso lacy | No vessels | Absent | Absent | FGP | HP | No | No | FGP |
| 49 | Same | Brown | Iso lacy | Brown | Spots | Ov/Tu/Br | FGP | Adenoma | No | Yes | FGP |
| 50 | Same | Same | Iso lacy | Iso lacy | Spots | Spots | FGP | HP | No | No | FGP |
| 51 | Brown | Brown | Brown | Brown | Ov/Tu/Br | Ov/Tu/Br | HP | AdenoCA | Yes | Yes | HP |
| 52 | Brown | Brown | Brown | Iso lacy | Ov/Tu/Br | Absent | HP | AdenoCA | Yes | No | HP |
| 53 | Brown | Brown | Brown | Brown | Ov/Tu/Br | Ov/Tu/Br | HP | AdenoCA | Yes | Yes | HP |
| 54 | Brown | Brown | Brown | Brown | Ov/Tu/Br | Ov/Tu/Br | HP | AdenoCA | Yes | Yes | HP |
| 55 | Same | Same | Iso lacy | Iso lacy | Spots | Spots | FGP | FGP | No | No | FGP |
| 56 | Same | Brown | Brown | Brown | Spots | Ov/Tu/Br | HP | Adenoma | Yes | Yes | HP |
| 57 | Same | Same | Iso lacy | Iso lacy | Absent | Absent | FGP | FGP | No | No | FGP |
| 58 | Same | Same | Iso lacy | Iso lacy | Absent | Absent | FGP | FGP | No | No | FGP |
| 59 | Same | Brown | Iso lacy | Brown | Spots | Absent | FGP | FGP | No | No | FGP |
| 60 | Same | Brown | No vessels | Brown | Ov/Tu/Br | Ov/Tu/Br | HP | Adenoma | Yes | Yes | HP |
| 61 | Same | Same | Iso lacy | Iso lacy | Absent | Absent | FGP | FGP | No | No | FGP |
| 62 | Same | Same | Brown | Brown | Ov/Tu/Br | Ov/Tu/Br | HP | HP | Yes | Yes | HP |
| 63 | Same | Brown | Iso lacy | Iso lacy | Absent | Absent | FGP | FGP | No | No | FGP |
| 64 | Brown | Brown | Iso lacy | Brown | Ov/Tu/Br | Ov/Tu/Br | Adenoma | Adenoma | No | Yes | HP |
| 65 | Same | Same | No vessels | No vessels | Spots | Absent | FGP | FGP | Yes | No | FGP |
| 66 | Same | Brown | Iso lacy | Brown | Absent | Ov/Tu/Br | FGP | Adenoma | No | Yes | FGP |
| 67 | Same | Same | No vessels | No vessels | Spots | Absent | FGP | HP | Yes | No | FGP |
| 68 | Brown | Brown | Brown | Brown | Ov/Tu/Br | Ov/Tu/Br | Adenoma | Adenoma | Yes | Yes | HP |
| 69 | Same | Brown | No vessels | Brown | Absent | Absent | FGP | Adenoma | No | No | FGP |
| 70 | Same | Brown | Iso lacy | Iso lacy | Spots | Absent | FGP | HP | No | No | FGP |
| 71 | Same | Brown | No vessels | No vessels | Spots | Spots | HP | Adenoma | Yes | Yes | FGP |
| 72 | Brown | Brown | Brown | Brown | Ov/Tu/Br | Ov/Tu/Br | HP | Adenoma | Yes | Yes | HP |
| 73 | Same | Brown | Brown | Brown | Ov/Tu/Br | Ov/Tu/Br | HP | Adenoma | Yes | Yes | HP |

Color: Same or lighter than background (Same), Brown relative to background (Brown)

Vessel pattern: No vessels, Isolated lacy vessels (Iso lacy), Brown vessels surrounding white structures (Brown)

Surface pattern: Dark or white spots of uniform size (Spots); Homogenous absence of pattern (Absent); Oval, tubular, or branched white structures (Ov/Tu/Br).

Prediction: Overall prediction by endoscopist of histology of polyp. FGP, Fundic gland polyp. HP, Hyperplastic polyp. AdenoCA, Adenocarcinoma.

Biopsy: Final Algorithm--Do not biopsy if either isolated lacy vessel pattern or homogeneous absence of surface pattern; biopsy all others.
